# Improved production of 2′-fucosyllactose in engineered *Saccharomyces cerevisiae* expressing a putative α-1, 2-fucosyltransferase from *Bacillus cereus*

**DOI:** 10.1186/s12934-021-01657-5

**Published:** 2021-08-23

**Authors:** Mingyuan Xu, Xiangfeng Meng, Weixin Zhang, Yu Shen, Weifeng Liu

**Affiliations:** State Key Laboratory of Microbial Technology, Microbial Technology Institute, Shandong University, No.72 Binhai Road, Qingdao, 266237 People’s Republic of China

**Keywords:** 2′-fucosyllactose, α-1, 2-fucosyltransferase, *Bacillus cereus*, *Saccharomyces cerevisiae*, GDP-l-fucose

## Abstract

**Background:**

2′-fucosyllactose (2′-FL) is one of the most abundant oligosaccharides in human milk. It constitutes an authorized functional additive to improve infant nutrition and health in manufactured infant formulations. As a result, a cost-effective method for mass production of 2′-FL is highly desirable.

**Results:**

A microbial cell factory for 2′-FL production was constructed in *Saccharomyces cerevisiae* by expressing a putative α-1, 2-fucosyltransferase from *Bacillus cereus* (FutBc) and enhancing the *de novo* GDP-l-fucose biosynthesis. When enabled lactose uptake, this system produced 2.54 g/L of 2′-FL with a batch flask cultivation using galactose as inducer and carbon source, representing a 1.8-fold increase compared with the commonly used α-1, 2-fucosyltransferase from *Helicobacter pylori* (FutC). The production of 2′-FL was further increased to 3.45 g/L by fortifying GDP-mannose synthesis. Further deleting *gal80* enabled the engineered strain to produce 26.63 g/L of 2′-FL with a yield of 0.85 mol/mol from lactose with sucrose as a carbon source in a fed-batch fermentation.

**Conclusion:**

FutBc combined with the other reported engineering strategies holds great potential for developing commercial scale processes for economic 2′-FL production using a food-grade microbial cell factory.

**Supplementary Information:**

The online version contains supplementary material available at 10.1186/s12934-021-01657-5.

## Background

Human milk oligosaccharides (HMOs) constitute a large family of oligosaccharides that are abundant only in human milk (5–15 g/L) and are believed to be critical for infant health [1]. In addition to their probiotic effect based on their ability to selectively stimulate the growth of Bifidobacteria (bifidus factor) [2], HMOs benefit the health of newborns in several other ways, including modulating immune responses, preventing pathogen adhesion to intestinal cells and providing sialic acid for infant brain development [3–5]. Among the various HMOs, 2′-fucosyllactose (Fucα1-2Galβ1-4Glc, 2′-FL, 2 g/L) is one of the key functional oligosaccharides in human breast milk, accounting for up to 30% of total HMOs [1]. As a main HMO constituent, 2′-FL has been considered to be a promising infant formula additive as well as in food due to its various beneficial effects on infant health. Moreover, its use has been approved by U.S. Food and Drug Administration (FDA) and European Food Safety Authority (EFSA) [6]. Intensive efforts have thus been made to develop microbial cell factories for economic production of 2′-FL on a large scale [2].

Among the various strategies developed to synthesize 2′-FL, whole-cell synthesis of 2′-FL using genetically engineered microorganisms represents a promising alternative with clear advantages over enzyme-catalyzed or chemo-enzymatic synthesis [7–9]. To achieve 2′-FL production in relevant microorganism strains, an ample supply of two key precursors is required, i.e., lactose and guanosine 5′‑diphosphate‑L‑fucose (GDP-l-fucose). While lactose is relatively inexpensive and can be provided in a feeding strategy, GDP-l-fucose can be derived from either the *de novo* pathway or the *salvage* pathway. The *salvage* pathway involves the intracellular conversion of exogenous l-fucose to fucose-1-phosphate by l-fucose kinase and further to GDP-l-fucose by fucose-1-phosphate guanylyltransferase [10]. A bifunctional enzyme l-fucokinase/GDP-l-fucose phosphorylase (Fkp) has been reported to catalyze these two reactions and has been employed for 2′-FL microbial cell factory construction [11–14]. In the *de novo* pathway, GDP-l-fucose is generated via fructose-6-phosphate and GDP-mannose, which is then metabolically converted to GDP-l-fucose by GDP-mannose-4, 6-dehydrogenase (Gmd) and GDP-4-keto-6-deoxymannose-3, 5-epimerase-4-reductase (WcaG) [15]. As the final step in 2′-FL biosynthesis, the fucosyl residue from the donor substrate (GDP-l-fucose) is transferred onto the acceptor substrate (lactose) catalyzed by α-1,2-fucosyltransferase (α-1, 2-FT) [8].

Whereas the most common cell factory platform for 2′-FL production reported so far is *Escherichia coli* [12–14, 16, 17], attempts have been also made in a few other microorganisms including *Bacillus subtilis* and *Saccharomyces cerevisiae* (Table 1). The latter microorganisms do not cause the specific concerns of endotoxin carryover as occurs with engineered *E. coli* [18–21]. In addition to being in its generally recognized as safe (GRAS) status, *S. cerevisiae* also has advantages over bacteria in not being susceptible to phage infections during fermentation as well as its rich intracellular pool of GDP-mannose, which make it a preferred production platform for 2′-FL [22]. However, currently reported efforts to engineer *S. cerevisiae* using either the *salvage* pathway or the *de novo* pathway have resulted in low 2′-FL titers (0.50 g/L and 0.51 g/L, respectively) in batch culture. Although a titer of 15 g/L was achieved by an engineered *S. cerevisiae* strain in a fed-batch culture, 2′-FL synthesis was only induced on addition of copper sulfate [19–21]. Recently, an engineered *S. cerevisiae* strain capable of assimilating xylose has been reported to produce 25.5 g/L of 2′-FL from a mixture of xylose and lactose in a fed-batch fermentation [23].

**Table 1:** 2′-FL production in different engineered organisms

Organism	Strategy	Titer	Yield	References
*E. coli*	*de novo* and *salvage* pathway; FutC from *H. pylori*	20.28 ± 0.83 g/L	309.16 ± 12.61 mg/g CDW	[12]
*E. coli*	*salvage* pathway; FutC from *H. pylori*, FucI is deleted	23.1g/L	0.37 mole/mole lactose	[14]
*E. coli*	*salvage* pathway; WcfB from *Bacteroides fragilis*	15.4 g/L	0.858 g/g lactose	[17]
*E. coli*	*de novo* pathway; FutC from *H. pylori*; deleting Ion and WcaJ; overexpressing Zwf and PntAB	9.12 g/L	Data not shown	[16]
*E. coli*	*salvage* pathway; FutC from *H. pylori*; deleting FucI, araA and rhaA	47.0 g/L	0.52mole 2-FL/mole fucose	[13]
*E. coli*	*de novo* pathway; α-1, 2-FT from *Thermosynechococcus elongatus*	0.49 g/L	Data not shown	[28]
*B. subtilis*	*salvage* pathway; FutC from *H. pylori*; enhancing GDP regeneration	5.01 g/L	0.85 mol/mol fucose	[18]
*S. cerevisiae*	*salvage* pathway; FutC from *H. pylori*	503 mg/L	0.44 mol/mol from l-fucose, 0.25 mol/mol from lactose	[21]
*S. cerevisiae*	*de novo* pathway; FutC from *H. pylori*	0.51 g/L	0.229 mol/mol from lactose	[19]
*S. cerevisiae*	*de novo* pathway; FutC from *H. pylori*; SUMO-tagged-FutC; CDT2 transporter	15 g/L	~ 0.156 g/g CDW	[20]
*S. cerevisiae*	*de novo* pathway; WbgL from *E. coli*; utilizing xylose; Extra copies of 2′-FL synthetic genes	25.5 g/L	0.82 mol/mol from lactose	[23]
*S. cerevisiae*	*de novo* pathway; FutBc from *B. cereus*; overexpressing Sec53 and Psa1.	26.63 g/L	0.85 mol/mol from lactose	This study

Among the various factors that likely contribute to 2′-FL production, the catalytic performance and efficient expression of α-1, 2-FT are reasonably considered to be crucial for the 2′-FL biosynthesis pathway due to the low reported activity of known α-1, 2-FTs [9]. Specifically, most of the biochemically characterized α-1, 2-FTs including *H. pylori* FutC and *E. coli* O126 WbgL display very low specific activity of < 1 U/mg although several reports have successfully employed these enzymes for 2′-FL synthesis [24–27]. Nonetheless, recent efforts have identified other natural diverse sources of α-1, 2-FTs [16, 17, 28]. The search for α-1, 2-FTs with sufficient activity in the production strain is critical not only for catalyzing the effective fucosyl transfer onto the acceptor lactose, but also for keeping GDP-l-fucose concentration at an appropriate level to avoid feedback inhibition of flux channeling GDP-mannose to GDP-l-fucose.

**Fig. 1:**
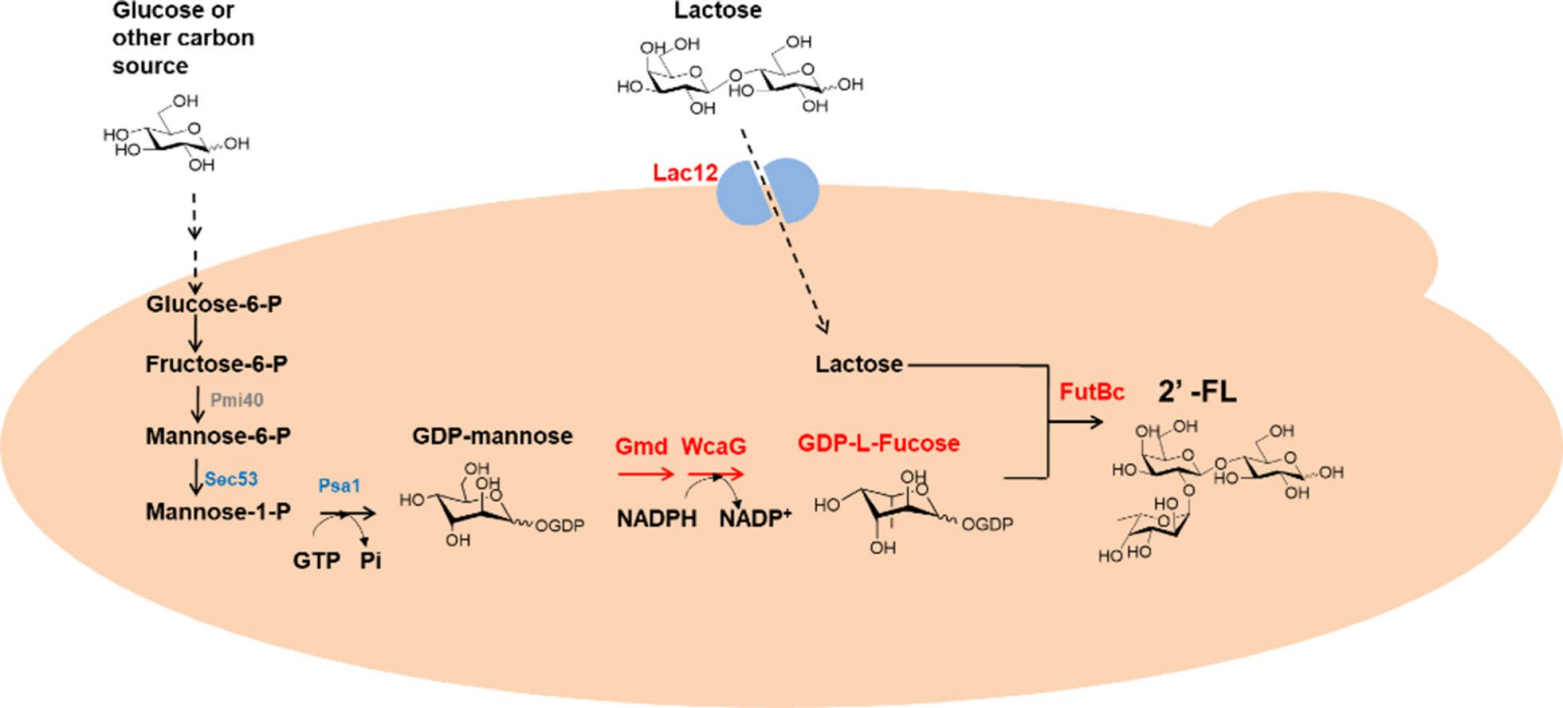
2′-FL biosynthesis and production optimization in *S. cerevisiae*. Lac12, Gmd, WcaG and α-1, 2-FT were heterologously overexpressed in *S. cerevisiae* for the biosynthesis of 2′-FL. Pmi40 and Sec53 were overexpressed to improve 2′-FL production by enhancing the GDP-mannose supply. Full names of enzymes in the figure: Lac12: Lactose permease; Gmd: GDP-mannose-4, 6-dehydrogenase; WcaG: GDP-4-keto-6-deoxymannose-3, 5-epimerase-4-reductase; FutBc: α-1, 2-fucosyltransferase; Pmi40: mannose-6-phosphate isomerase; Sec53: phosphomannomutase; Psa1: GDP-mannose pyrophosphorylase

In this study, we described an engineering strategy for 2′-FL synthesis in *S. cerevisiae* by enabling lactose uptake in combination with GDP-l-fucose production via the *de novo* pathway (Fig. 1). 2′-FL synthesis was achieved by introducing different microbial α-1, 2-FTs in the engineered strain with a putative α-1, 2-FT from *Bacillus cereus* (FutBc) resulting in 2′-FL titers as high as 2.54 g/L. The production of 2′-FL was further improved by enhancing GDP-mannose flux. Finally, deleting *gal80* in the engineered strain enabled the production of 26.63 g/L of 2′-FL from sucrose in a fed-batch fermentation.

### Results

### **Engineering*S. cerevisiae*for 2′-FL production**

*S. cerevisiae* lacks the native capacity to catabolize lactose, which is a desirable characteristic for 2′-FL synthesis since no competitive consumption of the acceptor substrate occurs. On the other hand, *S. cerevisiae* is incapable of lactose uptake and requires a heterologous lactose transporter to accumulate lactose intracellularly. We therefore chose to integrate *Kluyveryomyces lactis* lactose permease encoding gene *lac12* under the control of the inducible *gal1* promoter into the genome of *S. cerevisiae* W303-1a, resulting in strain FL01. Incubating pre-induced FL01with galactose in YP medium with 0.4% (w/v) lactose for 15 min led to intracellular lactose accumulation in contrast with no accumulation in the parental strain (Additional file 1: Figure S1A). To further confirm the functionality of the introduced Lac12, a plasmid expressing *K. lactis* β-galactosidase Lac4 was simultaneously introduced in FL01. The resultant strain (FL02) was able to grow on lactose as the sole carbon source after being pre-cultured on galactose while wild type W303-1a could not (Additional file 1: Figure S1B).

**Fig. 2:**
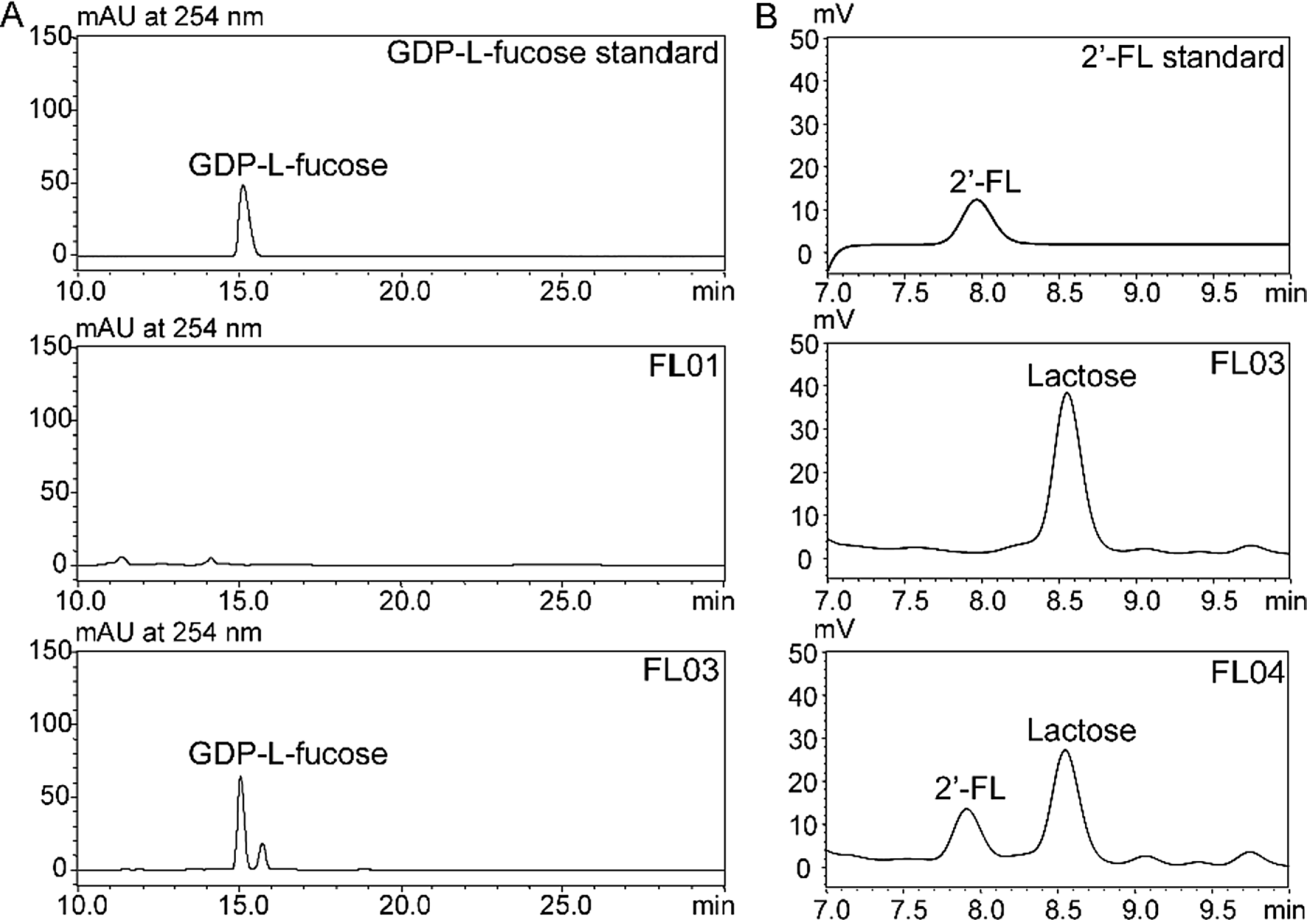
Production of GDP-l-fucose (**A**) and 2′-FL (**B**) by the engineered strains. To produce GDP-l-fucose, strains FL01 and FL03 were cultured in YP medium containing 2% (w/v) glucose and 2% (w/v) galactose for 48 h. To produce 2′-FL, strain FL04 expressing FutC from *H. pylori* and the control strain FL03 were cultured in YP medium containing glucose 2% (w/v) and 0.12 g/L adenine. Galactose 3% (w/v) and lactose 0.2% (w/v) were added at 24 h and cultured for another 48 h. 2′-FL were quantified by HPLC

To enable the *de novo* production of the precursor GDP-l-fucose, Gmd and WcaG encoding genes from *E. coli* were expressed under the control of the bidirectional *gal1-10* promoters in strain FL01 to obtain the FL03 strain. Consistent with previous reports [19], FL03 accumulated 5.8 mg/L GDP-l-fucose after being cultured on 2% (w/v) glucose and 2% (w/v) galactose for 48 h while no GDP-l-fucose was detected in FL01 (Fig. 2A). To accomplish 2′-FL biosynthesis, α-1, 2-FT from *H. pylori* (FutC) which has been shown to be one of the most efficient α-1, 2-FTs in catalyzing the fucosyl transfer to lactose, was further overexpressed in FL03 under the *gal1* promoter [12, 16, 18]. When the resulting FL04 strain was cultured in YP medium containing 2 g/L lactose for 72 h, the extracellular 2′-FL reached 1.18 g/L and the total 2′-FL measured after cell lysis was 1.41 g/L (Fig. 2B). The yield of total 2′-FL from lactose was 0.50 mol/mol.

### **Improving 2′-FL biosynthesis using a putative α-1, 2- FT from*B. cereus***

Considering that the low catalytic efficiency of α-1, 2-FT constitutes a limiting factor in 2′-FL production, we elected to explore other α-1, 2-FTs from various microorganisms in an attempt to enhance 2′-FL biosynthesis. FucT2 from *Homo sapiens* and FutC from *H. pylori* were thus used as the query sequence for protein-BLAST searches for α-1, 2-FTs by excluding close homologs from the same genus. Specifically, we selected putative α-1, 2-FTs from gut microorganisms and some known probiotic bacteria including *Acetobacter* sp. (FutAs), *B. cereus* (FutBc), *Bacteroides eggerthii* (FutBe), *Bacteroides unifomis* (FutBu) and *Neocallimastix californiae* (FutNc). Amino acid sequence alignment of the selected sequences revealed that all the putative α-1, 2-FTs belonged to the glycotransferase family 11 (GT11) since they possessed motifs I to IV along with conserved catalytic residues (Additional file 1: Fig. S3B). However, they displayed relatively low aa sequence identity to FutC from *H. pylori* (29–37%) and FucT2 from *H. sapiens* (17–25%) (Additional file 1: Table S2). Phylogenetic analysis showed that FutNc and FutAs were more closely related to FutC whereas FutBc, FutBu and FutBe formed an independent lineage that clustered together with the biochemically characterized FutTe from *Thermosynechococcus elongates* and FutN from *Bacteroides vulgatus* (Additional file 1: Figure S2A) [16, 28].

The above enzyme encoding genes were individually expressed under the *gal1* promoter in FL03 to evaluate their ability to catalyze the synthesis of 2′-FL. Among the α-1, 2- FT homologs tested, 2′-FL was produced with FutAs (FL05) and FutBe (FL07), reaching 37% and 63% of that of FutC (FL04), respectively. Notably, expression of FutBc significantly enhanced 2′-FL production with a total titer of 2.54 g/L in the resulting FL06 strain (Additional file 1: Fig. 3A and Additional file 1: Figure S3), which was 1.8-fold that of FutC, with a volumetric productivity of 0.035 g/L/h and a yield of 0.89 mol/mol from lactose. Extracellular 2′-FL accounted for 83%, 69%, 86% and 89% of the total 2′-FL produced in strains FL04, FL05, FL06 and FL07, respectively.

**Fig. 3:**
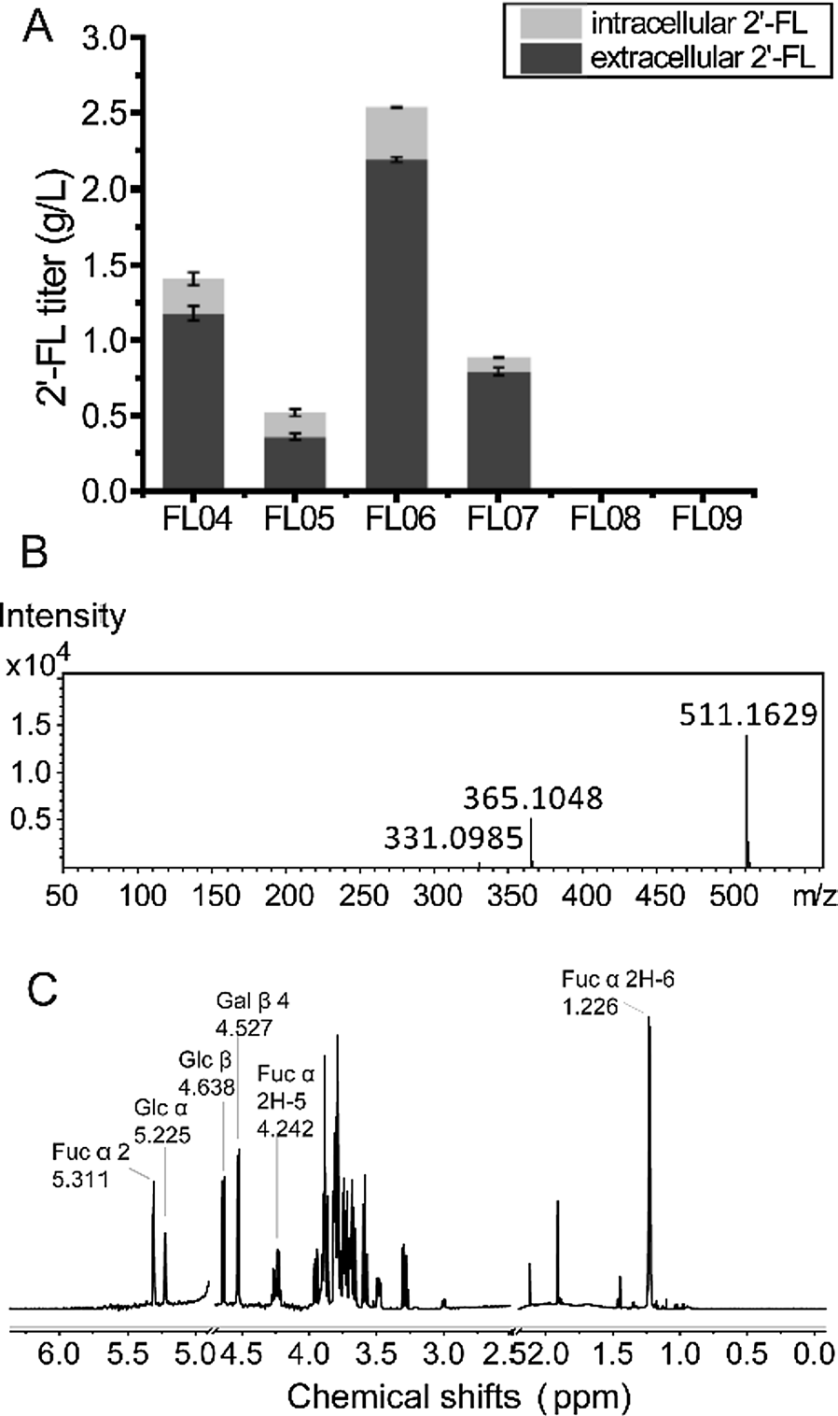
Enhanced production of 2′-FL by putative α-1, 2-FT from *B. cereus*. **A** Production of 2′-FL by engineered *S. cerevisiae* with different α-1, 2-FTs. Strains expressing α-1, 2-FTs from different organisms, including FutC from *H. pylori* (FL04) and selected α-1, 2-FTs from *Acetobacter* sp. (FL05), *B. cereus* (FL06), *B. eggerthii* (FL07), *B. unifomis* (FL08), and *N. californiae* (FL09) were cultured in YP medium containing glucose 2% (w/v) and 0.12 g/L adenine. Galactose 3% (w/v) and lactose 0.2% (w/v) were added at 24 h and cultured for another 48 h. 2′-FL were quantified by HPLC. **B** LC-MS analysis of 2′-FL produced by FL06. **C** ^1^H-NMR spectrum of 2′-FL purified from FL06 fermentation broth

To verify the production of 2′-FL in FL06, analysis of cultures by LC-MS revealed a peak with the same retention time and *m/z* as the 2′-FL standard (511.16 [M + Na]^+^ ; Fig. 3B). Moreover, the ^1^H NMR spectrum of the FutBc synthesized 2′-FL was shown to match that of a reported 2′-FL standard [29]. Specifically, typical ^1^H chemical shift values for α and β anomers of reducing glucose unit were detected at δ 5.225 and δ 4.638, respectively. The anomeric signals at δ 5.311 and δ 4.527 corresponded to the presence of fucosyl-(α1→2) unit and galactosyl-(β1→4) unit, respectively. The chemical shift of H-5 and H-6 of fucosyl-(α1→2) unit were detected at δ 4.242 and δ 1.226, respectively (Fig. 3C). Together these analyses confirmed 2′-FL production by the engineered *S. cerevisiae* expressing FutBc.

### Enhancing 2′-FL production by strengthening GDP-mannose supply

Strain FL06 was selected for further metabolic engineering to improve 2′-FL production. Firstly, the initial lactose concentration effects on 2′-FL production were evaluated. Notably, while decreasing the initial lactose concentration from 0.2% (w/v) to 0.1% (w/v) reduced 2′-FL production by 46%, there was no improvement in 2′-FL titers when the initial lactose concentration was increased to 0.4% (w/v) (Additional file 1: Figure S4A). It is worth noting that the volumetric GDP-l-fucose concentration dramatically decreased in yeast cultured on 0.2% lactose compared with 0.1% lactose, but no further decreases occurred with 0.4% lactose (Additional file 1: Figure S4B), implying that lower concentrations of GDP-l-fucose might not support the efficient transfer of fucose to lactose for 2′-FL synthesis.

**Fig. 4:**
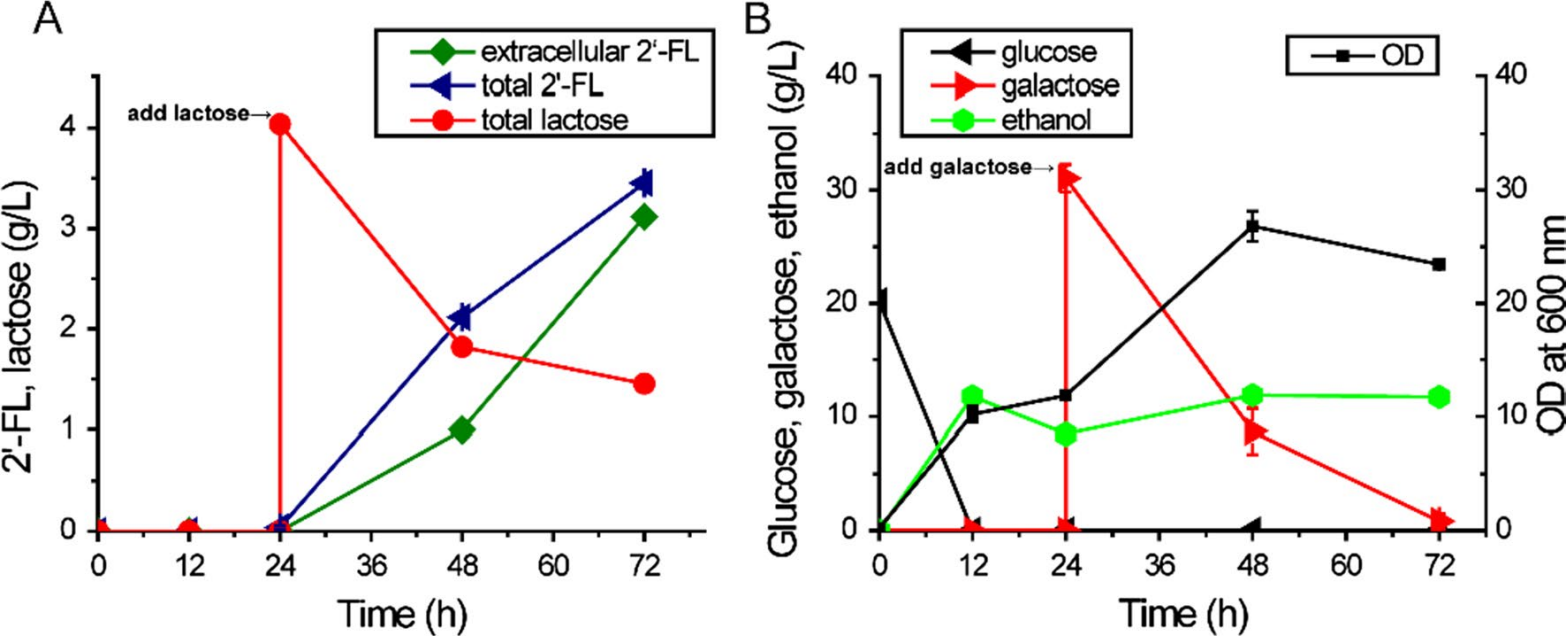
Fermentation profiles of strain FL16 with enhancing GDP-mannose supply for improved 2′-FL production. **A** Concentration of total and extracellular 2′-FL, and Lactose during fermentation; **B** Concentration of glucose, galactose, ethanol, and cell density (OD_600nm_) during fermentation. The engineered strains FL16 were cultured in YP medium containing glucose 2% (w/v) and 0.12 g/L adenine. Galactose 3% (w/v) and lactose 0.4% (w/v) were added at 24 h and cultured for another 48 h

As GDP-l-fucose is derived from GDP-mannose, enhancing the biosynthesis of GDP-mannose could benefit 2‘-FL production. GDP-mannose is synthesized from fructose-6-phosphate by three sequential reactions catalyzed by mannose-6-phosphate isomerase (Pmi40 in *S. cerevisiae*), phosphomannomutase (Sec53 in *S. cerevisiae*) and GDP-mannose pyrophosphorylase (Psa1 in *S. cerevisiae*), respectively. On this basis we chose to investigate the impact of strengthening the carbon flux from fructose-6-phosphate to GDP-mannose on 2′-FL production. Sec53 and Psa1 were thus overexpressed in FL06, resulting in strain FL16. Batch fermentation analysis of FL16 in YP medium showed 20 g/L of glucose was consumed within 12 h. 2′-FL production was initiated by adding 3% galactose and 0.4% lactose at 24 h, and 2′-FL accumulated to 3.45 g/L at 72 h with a yield of 0.61 mol/mol from total lactose. At this time point, while the initially added galactose was completely consumed, about 33% of lactose was left unassimilated and a relative constant level of ethanol up to 11.27 g/L was also produced after glucose depletion and during 2′-FL synthesis (Fig. 4A). Of note, a slight but significant drop in cell densities (OD_600_ 26.8 to 23.4) was observed from 48 h to 72, which was further decreased thereafter (Fig. 4B and data not shown). This decrease in cell densities was accompanied by a change in the ratio of extracellular/total 2′-FL from 47.3% at 48 h to 90.2% at 72 h (Fig. 4A). To evaluate the possibility that the yeast cell walls might have become more fragile and permeable, their sensitivity to sodium dodecyl sulfate (SDS) was tested (Additional file 1: Figure S5). Instructively, yeast cells with higher capability of 2′-FL synthesis displayed increased sensitivity to SDS treatment, implicating that cell wall integrity was somehow compromised. Lastly we found there was only a marginal benefit to 2′-FL production using a strategy of further overexpressing Pmi40 and an mitochondria NADH kinase Pos5 in cytoplasm to improve the cytosolic NADPH supply (Additional file 1: Figure S6).

### 2′-FL production in fed-batch fermentation

**Fig. 5:**
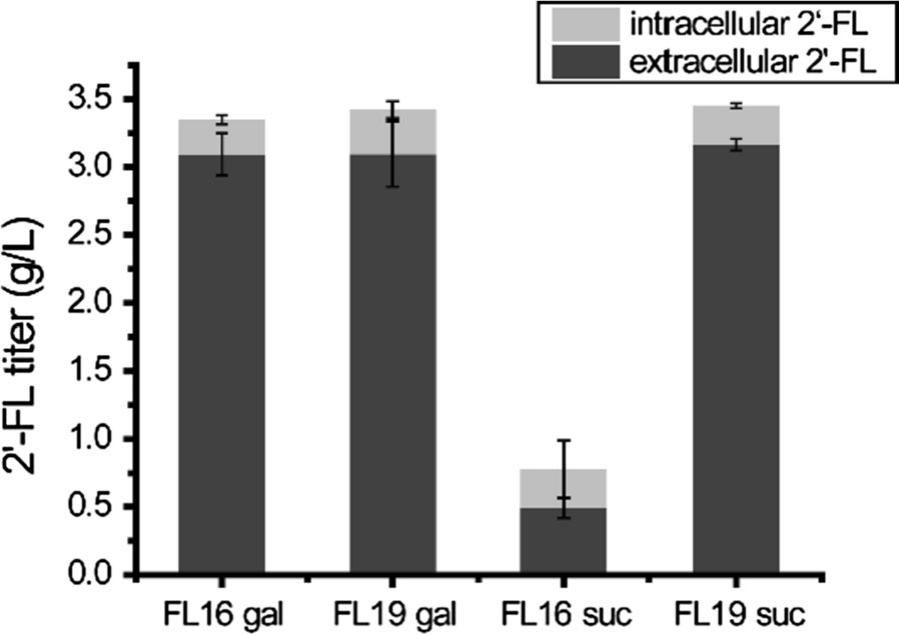
Deletion of Gal80 enabled 2′-FL production with sucrose as the carbon source. Gal80 was knockout in FL16, resulting strain FL19. The strains were cultured in YP medium containing glucose 2% (w/v) and 0.12 g/L adenine. Galactose 3% (w/v) or sucrose 3% (w/v) and lactose 0.4% (w/v) were added at 24 h and cultured for another 48 h. FL16 gal and FL19 gal indicate 2′-FL production from galactose while FL16 suc and FL19 suc indicate 2′-FL production from sucrose

**Fig. 6:**
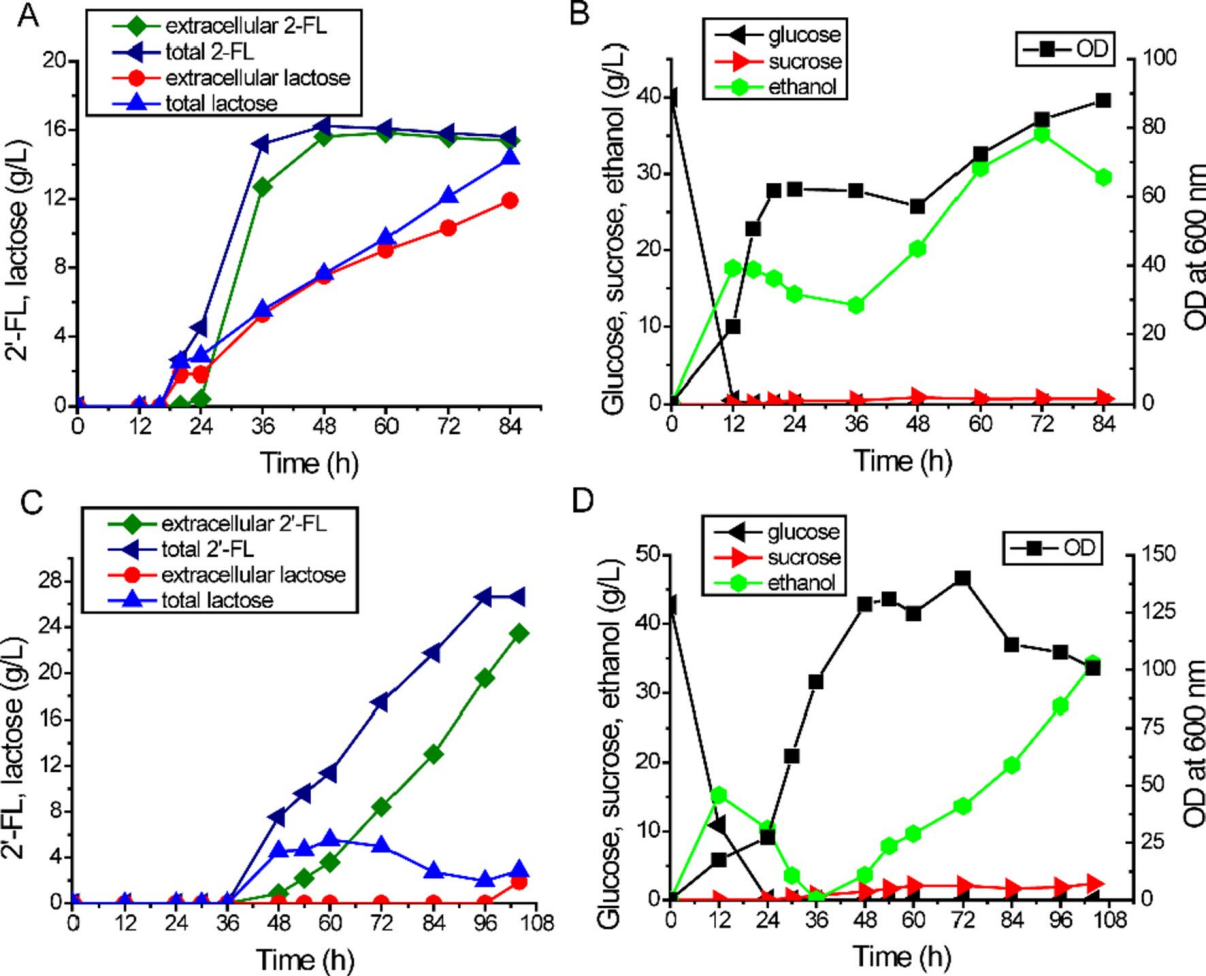
2′-FL production by FL19 in fed-batch fermentation. **A** Concentration of 2′-FL and lactose during fermentation in YP medium. **B** Concentration of glucose, sucrose, ethanol, and cell density (OD_600nm_) during fermentation in YP medium. **C** Concentration of 2′-FL and Lactose during fermentation in Verduyn medium. **D** Concentration of glucose, sucrose, ethanol, and cell density (OD_600nm_) during fermentation in Verduyn medium

Our engineering strategies in *S. cerevisiae* employed galactose inducing promoters (*P*_*gal*1_, *P*_*gal10*_) for the overexpression of 2′-FL biosynthetic pathway genes. However, considering that galactose is expensive, its use in industrial production applications is limited for economic reasons. Thus, we evaluated 2′-FL synthesis using alternative carbon sources. Since *gal80* is the major transcriptional repressor of galactose-inducible gene promoters [30, 31], *gal80* was therefore further deleted in FL16. This should allow the expression of the *gal* promoter-controlled 2′-FL pathway genes on carbon sources such as sucrose that has been shown to capable of minimizing the catabolite repression from glucose [31]. The resulting strain FL19 produced 3.45 g/L 2′-FL on 30 g/L sucrose and 4 g/L lactose, while FL16 only produced 0.78 g/L 2′-FL under the same conditions (Fig. 5). To increase the titer of 2′-FL and test the capability of the engineered strain in a large-scale production setting, we examined the performance of FL19 in a fed-batch 5 L bioreactor with feeding sucrose and lactose to maximize cell growth and 2′-FL production. Feeding of lactose and sucrose was started at 16 h after glucose depletion to initiate 2′-FL synthesis and the concentration of sucrose in the broth was maintained at relative low level during fermentation (< 0.9 g/L). The production of 2′-FL reached 16.23 g/L (15.63 g/L extracellularly and 0.60 g/L intracellularly) at 48 h with a volumetric productivity of 0.34 g/L/h (Fig. 6A). The final yield of 2′-FL from total lactose was 0.60 mol/mol at 48 h. No more 2′-FL was accumulated after 48 h although cell densities (OD_600nm_) increased from 57.20 to 88.00 at 84 h with ethanol concentration being increased from 19.66 g/L to 29.88 g/L (Fig. 6B).

We also conducted a fed-batch fermentation using Verduyn medium that has been reported to allow for higher yeast cell densities and potentially higher production of 2′-FL. Lactose feeding was initiated at 36 h when a cell density reached OD ~ 90. The FL19 strain produced a total of 26.63 g/L 2′-FL at 96 h (19.56 g/L extracellular and 7.07 g/L intracellular) (Fig. 6C). The overall volumetric productivities were 0.28 g/L/h and 0.44 g/L/h for the whole fermentation process and production phase, respectively. The molar conversion rate of lactose to 2′-FL was 0.85 mol/mol.

## Discussion

*S. cerevisiae* is a proven GRAS host for 2′-FL production [19–21, 23]. but the feasibility of using these engineered yeasts is affected by the comparatively lower titers of 2′-FL compared to those generated in engineered *E. coli* [13, 16, 20]. Most recently, an engineered *S. cerevisiae* strain was reported to achieve 25.5 g/L 2′-FL with xylose as a co-substrate in a fed-batch fermentation [23]. Although the titers from our study are still less than those produced in *E. coli* (47.0 g/L) fed with lactose and fucose [13], this outcome currently represents the highest titer reported from *S. cerevisiae*.

Among previous reports utilizing *S. cerevisiae*, *H. pylori* FutC has been shown to produce the highest amount of 2′-FL among the α-1, 2-FTs known to perform well for 2′-FL production in *E. coli* [16, 17, 20]. To improve 2′-FL production in an *S. cerevisiae* context, we used database mining to identify potential alternative α-1, 2-FT genes from various microorganisms. Despite its relatively low sequence identity to other known α-1, 2-FTs, we found that FutBc from *B. cereus* achieved significantly higher 2′-FL titers. The enhanced production could be attributed to a higher expression level of active forms of FutBc than FutC in *S. cerevisiae* although its potentially higher catalytic activity cannot be excluded. Unfortunately, attempts to verify FutBc activity in vitro using fucosylation assays with recombinant enzyme from *E. coli* failed (data not shown). Although the precise reason for the inactivity remains unclear, similar results have been previously shown where WcfB purified from *E. coli* did not accept lactose as substrate in vitro even though the enzyme produced 2′-FL in vivo [17]. Notably, Western blot assays revealed a 3-fold higher expression level of FutBc compared to FutC (Additional file 1: Figure S3), suggesting high expression levels are important. Regardless, our study successfully identified a highly effective α-1, 2-FT from *B. cereus*, which may serve as a starting point to be improved further by directed evolution to enable efficient 2′-FL production in yeast.

As the donor substrate, GDP-l-fucose supply constitutes another limiting factor for the efficient synthesis of 2′-FL. The *de novo* synthesis of GDP-l-fucose in *S. cerevisiae* has been achieved in previous studies by introducing Gmd and WcaG enzymes from *E. coli* [19, 32]. However, no further optimization has been performed to enhance the supply of GDP-l-fucose. In *E. coli*, overexpressing both ManB (Sec53 in *S. cerevisiae*) and ManC (Psa1 in *S. cerevisiae*) together increased the yield of GDP-l-fucose, while expressing only ManB or ManC had no effect [33]. Although *S. cerevisiae* is thought to produce abundant amounts of GDP-mannose, the majority is consumed for cell wall biosynthesis in order to maintain cellular integrity. To ensure the ample supply of GDP-l-fucose for 2′-FL synthesis, we chose to strengthen the GDP-mannose biosynthetic pathway by overexpressing of *sec53* and *psa1* in the *de novo* GDP-mannose synthesis pathway. This approach demonstrated a clear increase in 2′-FL synthesis, suggesting a beneficial effect of enhancing GDP-mannose synthesis. On top of *sec53* and *psa1* overexpression, overexpressing *pmi40* which encodes the enzyme that catalyzes the reciprocal reaction between fructose-6-P and mannose-6-P only led to a slight increase in 2′-FL synthesis (Additional file 1: Figure S6), which is consistent with results in *E. coli* [10].

Considering that Gmd catalyzes the conversion of GDP-4-keto-6-deoxymannose to GDP-l-fucose with NADPH as a co-factor, NADPH levels have been also shown to be critical for the *de novo* synthesis of GDP-l-fucose and hence the production of 2′-FL. A significant increase in 2′-FL (2.64 g/L to 4.81 g/L) has thus been observed when glucose-6-phosphate dehydrogenase (Zwf) of the pentose phosphate pathway (PPP) was overexpressed in *E. coli* [16]. However, our present study showed that purposely expressing the mitochondria NADH kinase Pos5 in the cytosol only slightly increased 2′-FL production (Additional file 1: Figure S6) [16]. Other strategies to expand the cytosolic NADPH pool, including Zwf overexpression, engineering the tricarboxylic acid (TCA) cycle by overexpressing isocitrate dehydrogenase Icd, or strengthening the anaplerotic pathway by overexpressing NADP^+^-dependent malate dehydrogenase MaeB, also merits further evaluation [34–36].

In the present study, the extracellular 2′-FL detected at the end of the fermentation accounted for 97% of the total 2′-FL in YP medium and 73% of the total 2′-FL produced in Verduyn medium, which is consistent with a report by Lee et al. [23]. Moreover, the ratio of extracellular to intracellular 2′-FL ratio in batch culture was similar to that reported by Liu et al. [19], but different from other reports showing that extracellular accumulation of 2′-FL accounted for only 25% of total production [20]. The reason for these discrepancies remains unclear but we did notice that the ratio of extracellular 2′-FL increased upon prolonged fermentation. Consistent with this phenomenon, we observed that yeast cells with higher 2′-FL production exhibited more fragile cell walls (Additional file 1: Figure S5). This observation was also made by Hollands et al. [20] and such fragility could conceivably account for the high extracellular ratio of 2′-FL observed in the present study. Moreover, given the role of GDP-mannose in cell wall biosynthesis, the compromised cell wall integrity likely resulted from the diversion of GDP-mannose to 2′-FL production, thus facilitating cell wall leakage.

## Conclusion

In conclusion, we successfully engineered *S. cerevisiae* for efficient production of 2′-FL (26.63 g/L in fed-batch fermentation) by employing a putative α-1, 2-FT from *B. cereus* and enhancing the biosynthesis of precursor substrate GDP-l-fucose. The identified *B. cereus* FutBc and the engineered *S. cerevisiae* strain in the present study thus represent valuable tools and a suitable microbial platform to further improve the production of 2′-FL.

## Methods

### Strains and culture conditions

*E. coli* DH5α used for plasmid construction and propagation was routinely cultured in Luria-Bertani (LB) broth at 37 °C with the corresponding antibiotics. *S. cerevisiae* W303-1a (*MATa ade2-1 can1-100 ura3-1 leu2-3,112 his3-11,15*) was used as the host for 2′-FL production. The yeast strains were cultured in YPD medium (peptone 2% (w/v), yeast extract 1% (w/v), glucose 2% (w/v) for batch fermentation or 4% (w/v) for fed-batch fermentation). The optimized Verduyn medium (glucose 4% (w/v), ammonium sulfate 1.5% (w/v), potassium dihydrogen phosphate 1.2% (w/v), magnesium sulfate heptahydrate 0.4% (w/v), 10 mL/L trace metal solution, 5ml/L vitamin solution, adenine sulfate 0.12% (w/v)) was used for fed-batch fermentation [20, 37]. Synthetic complete (SC) medium (yeast nitrogen base 0.17% (w/v), ammonium sulfate 0.5% (w/v), 2% glucose (w/v)) with appropriate amino acids were used for auxotroph selection of *S. cerevisiae* transformants. YP medium containing geneticin (G418) at a concentration of 250 mg/L was used for selecting transformant resistant to geneticin. YPD or SC solid medium were prepared by adding 15 g/L agar into the liquid media.

## Plasmids and strains construction

For the construction of strain FL01, *K. lactis lac12* (GenBank accession: X06997.1) was amplified by polymerase chain reactions (PCRs) from *K. lactis* genomic DNA. The expression cassette of *lac12* (P_*gal1*_-*lac12*-T_*cyc1*_) containing the *gal1* promoter, the *lac12* coding sequence and the *cyc1* terminator was assembled by fusion extension PCR and was cloned into the pRS304 plasmid at BamHI and XhoI sites, resulting in the pRS304-P_*gal1*_-*lac12*-T_*cyc1*_ plasmid, which was then linearized by Bsu36I before being transformed into W303-1a.

The Lac4 coding sequence was amplified from *K. lactis* genomic DNA and was similarly assembled with the *gal1* promoter and the *cyc1* terminator by fusion extension PCR to generate P_*gal1*_-*lac4*-T_*cyc1*_, which was then inserted between the XhoI and SpeI sites of the pRS425 plasmid using T5 exonuclease-dependent assembly to obtain the pRS425-P_*gal1*_-*lac4*-T_*cyc1*_ plasmid [38]. The resultant plasmid was transformed into the FL01 strain to obtain strain FL02.

For installing the *de novo* GDP-l-fucose synthesis pathway in *S. cerevisiae*, *gmd* and *wcaG* were amplified from *E. coli* K12 genomic DNA and assembled with the bidirectional *gal1-10* promoters using fusion extension PCR to put them under the control of these two promoters, respectively. The expression cassette was then inserted between SacI and KpnI sites in the pRS304 plasmid to obtain the pRS306-P_*gal1*_-*gmd-*T_*cyc1*_&P_*gal10*_*-wcaG-*T_*adh1*_ plasmid. The recombinant vector was linearized by NcoI before being transformed into FL01 to obtain the FL03 strain.

α-1, 2-FTs from various microorganisms were codon optimized for *S. cerevisiae* and synthesized by Genewiz (Suzhou, China), including those from *H. pylori* (GenBank accession: WP_080473865.1), *Acetobacter* sp. (GenBank accession: CDA17967.1), *B. cereus* (GenBank accession: EJR48924.1), *B. eggerthii* (GenBank accession: WP_004291980.1), *B. unifomis* (GenBank accession: WP_061412361.1), and *N. californiae* (GenBank accession: ORY35279.1). The synthesized coding sequences were PCR assembled with P_*gal1*_ and T_*cyc1*_ and the resultant expression cassettes were cloned into the pRS305 plasmid between the XbaI and HindIII sites. The respective recombinant vector was linearized by BspTI before being transformed into FL03, resulting in strains FL04, FL05, FL06, FL07, FL08 and FL09, respectively.

For enhancing GDP-mannose biosynthesis, *sec53* and *psa1* were amplified from *S. cerevisiae* W303-1a genomic DNA and assembled with the bidirectional *gal1-10* promoters amplified from pUMRI-A so that *sec53* expression was under the control of *gal1* and *psa1* was under the control of *gal10*, respectively. The resulting expression cassette was inserted between the XbaI and EcoRI sites of the pRS303 plasmid to obtain the pRS303-P_*gal1*_-*sec53-*T_*cyc1*_&P_*gal10*_*-psa1-*T_*adh1*_ plasmid, which was then linearized by BsiWI before being transformed into FL06 to generate strain FL16. For *pmi40* overexpression, the *pmi40* coding sequence was amplified and inserted between the BamHI and NheI sites of pUMRI-A to obtain the pUMRI-A-P_*gal10*_-*pmi40-*T_*adh1*_ plasmid, which was linearized by PciI before being transformed into FL16 to obtain FL17. To achieve *spos5* overexpression, the *pos5* coding sequence lacking the mitochondria targeting signal peptide was first amplified and inserted into pUMRI-A between EcoRI and SacI to obtain pUMRI-A-P_*gal1*_-*spos5-*T_*cyc1*_, and then *pmi40* was inserted into the pUMRI-A-P_*gal1*_-*spos5-*T_*cyc1*_ at the site of BamHI and NheI to generate pUMRI-A-P_*gal1*_-*spos5-*T_*cyc1*_&P_*gal10*_-*pmi40-*T_*adh1*_. The constructed vector was linearized by PciI and then transformed into FL16 to obtain strain FL18.

To knockout *gal80*, a primer pairs containing 45 bp homologous arms upstream or downstream of *gal80* were designed to amplify *KanMX*. The obtained PCR product was transferred into FL16 to obtain strain FL19.

The integrative plasmids or cassettes for gene overexpression or knockout were transformed into *S. cerevisiae* W303-1a by LiAc/SS carrier DNA/PEG method as described by Gietz et al. [39]. Transformants were selected on SC auxotrophic plate or YPD agar plates with 250 µg/mL G418. The successful integration or deletion of the target gene in selected transformants was confirmed by PCR. Primers used in this study were listed in Additional file 1: Table S1. All strains constructed in this study were listed in Table 2.

**Table 2:** Strains constructed in this study

Strain	Description	Reference or Source
FL01	W303-1a with pRS304-P_*gal1*_-*lac12*-T_*cyc1*_	This study
FL02	FL01 with pRS425-P_*gal1*_-*lac4*-T_*cyc1*_	This study
FL03	FL01 with pRS306-P_*gal1*_-*gmd-*T_*cyc1*_&P_*gal10*_*-wcaG-*T_*adh1*_	This study
FL04	FL03 with pRS305-P_*gal1*_-*futC* (from *H. pylori*)- T_*cyc1*_	This study
FL05	FL03 with pRS305-P_*gal1*_-*futAs* (from *Acetobacter* sp.)- T_*cyc1*_	This study
FL06	FL03 with pRS305-P_*gal1*_-*futBc* (from *B. cereus*)- T_*cyc1*_	This study
FL07	FL03 with pRS305-P_*gal1*_-*futBe* (from *B. eggerthii*)- T_*cyc1*_	This study
FL08	FL03 with pRS305-P_*gal1*_-*futBu* (from *B. unifomis*)- T_*cyc1*_	This study
FL09	FL03 with pRS305-P_*gal1*_-*futNc* (from *N. californiae*)- T_*cyc1*_	This study
FL16	FL06 with pRS303-P_*gal1*_-*sec53-*T_*cyc1*_&P_*gal10*_*-psa1-*T_*adh1*_	This study
FL17	FL16 with pUMRI-A-P*gal10*-*pmi40*-T_*adh1*_	This study
FL18	FL16 with pUMRI-A-P_*gal1*_-*spos5-*T_*cyc1*_&P_*gal10*_*-pmi40-*T_*adh1*_	This study
FL19	FL16 without Gal80	This study

### Yeast culture for 2′-FL production in shake flasks

Yeast strains were first cultured in liquid SC medium containing 2% glucose and lacking the appropriate amino acids (specifically, SC medium lacking tryptophan for FL01; tryptophan and leucine for FL02, tryptophan and uracil for FL03; tryptophan, uracil and leucine for FL04, FL05, FL06, FL07, FL08, FL09; tryptophan, uracil, leucine and histidine for FL16, FL17, FL18 and FL19) at 30 °C, 200 rpm for 12 h. Strains were then sub-cultured into 100 mL of YP medium with 2% (w/v) glucose and 0.12 g/L adenine at a starting OD_600nm_ of 0.1 in 500 mL flasks. Twenty-four hour after inoculum, galactose 3% (w/v) or sucrose 3% (w/v) and 0.2% (w/v) lactose (for strain FL04, FL05, FL06, FL07, FL08, FL09, FL10, FL11, FL12, FL13, FL14 and FL15) or 0.4% (w/v) lactose (for strain FL06, FL16, FL17, FL18 and FL19) were added and incubated at 30 °C with agitation at 240 rpm for another 48 h.

### Lactose transport analysis

To measure intracellular lactose, W303-1a and FL01 strains were cultured in liquid YP medium containing 2% (w/v) galactose for 24 h. The yeast cells were collected, washed twice with distilled water, and incubated with 0.4% (w/v) lactose in an equal volume of YP medium for 15 min. The cells were again collected and disrupted by glass beads in homogenizer (Precellys 24, Bertin) at 6000 rpm for 45 s, which was repeated for 5 times. The supernatant was obtained by centrifugation (10,000*×g*, 4 °C, 10 min) and used for TLC analysis. Samples of the supernatant (2 µL) were spotted on the TLC plates (Merck, silica gel 60 F254, 20 *× *20 cm). Glucose (1 mg/mL), galactose (1 mg/mL) and lactose (1 mg/mL) was used as the standard. The TLC plates were developed with 2-butanol: acetic acid: water (2:1:1, v/v/v) as a mobile phase and stained with orcinol/sulfuric acid staining reagent (0.1% oricinol w/v, 10% sulfuric acid v/v and 90% methanol v/v).

To further verify the function of Lac12, strains FL02 and W303-1a were cultured in YP medium with 2% (w/v) galactose overnight. The cells were collected, washed twice with water, and then transferred into equal volume of YP medium with 2% (w/v) lactose as the sole carbon source. The cell growth was monitored by measuring OD_600nm_ using a microplate reader (Infinite M200, Tecan).

### GDP-l-fucose production analysis

Strain FL03 was cultured in YP medium with 2% glucose (w/v) and 2% galactose (w/v) at 30 °C, 200 rpm for 48 h. Yeast cells from 5 mL culture were collected by centrifugation (5000*×g*, 5 min), washed twice with water, and were then resuspended in 1 mL distilled water to be disrupted by glass beads in homogenizer (Precellys 24, Bertin) at 6000 rpm for 45 s for 5 times. The supernatant was separated from the cell debris by centrifugation (13000*×g*, 10 min). and was resolved by a C18 column (ZORBAX Eclipse Plus C18 4.6 *×* 150 mm, Agilent) connected to a high-performance liquid chromatography (HPLC) system (Shimadzu, Kyoto, Japan) equipped with a UV detector. The column temperature was set at 30 °C. Trimethylamine (20 mM, pH 6.0) with 2% (v/v) acetonitrile was used as the mobile phase and the flow rate was set at 0.6 mL/min. GDP-l-fucose was detected at 254 nm.

### 2′-FL production analysis

One-milliliter culture was centrifuged to obtain the supernatant for quantification of the extracellular synthesized 2′-FL. To quantify the intracellular 2′-FL, the yeast cells were collected, washed, resuspended in 1 mL distilled water and disrupted by glass beads in homogenizer (6000 rpm for 45 s, repeated for 5 times). The cellular extracts were centrifuged at 13,300*×g* for 5 min. The resulting intracellular or extracellular supernatant (15 µL) was injected into HPLC system (Shimadzu, Kyoto, Japan) equipped with a RI detector and analyzed using the Rezex ROA-Organic Acid H ^+^ (8%) column (Phenomenex, Torrance, CA, USA) for the quantification of 2′-FL. The temperature of the column was set at 50 °C. H_2_SO_4_ (0.005 N) was used as the mobile phase and the flow rate was set at 0.6 mL/min.

### 2′-FL structural analysis

For mass spectrometry analysis of 2′-FL produced by FL06, the fermentation broth of FL06 was collected as described above. The extracellular sample was then diluted 50 times with distilled water and 1 µL of the diluted sample was injected into a LC-MS system (LTQ-Orbitrap velos pro ETD) connected to the Rezex ROA-Organic Acid H ^+^ (8%) column (Phenomenex, Torrance, CA, USA). The temperature of the column was set at 50 °C. The mobile phase used was 0.005 M formic acid and the flow rate was set at 0.6 mL/min.

For NMR analysis, extracellular 2′-FL produced by FL06 was precipitated by adding nine-fold volume of ethanol to the culture. After being stirred for 2 h, the precipitate was collected by centrifugation at 10,000*×g* for 10 min. The precipitated 2′-FL was resuspended in distilled water and was further purified by Bio-Gel P2 column (2.5 *×* 50 cm, BioRad) using 10 mM NH_4_HCO_3_ as eluent at a flow rate of 36 mL/h. The collected fractions were analyzed by TLC and those with 2′-FL only was combined and freeze-dried to obtain the purified 2′-FL. Prior to ^1^H NMR analysis, samples of 2′-FL were exchanged for three times in D_2_O (Cambridge Isotope Laboratories, Inc., MA, US) with intermediate lyophilization, which was then dissolved in 0.6 ml D_2_O. Chemical shifts (δ) are expressed in ppm by reference to internal acetone (δ 2.225 for ^1^H).

### Phylogenic analysis of α-1, 2-FTs

Phylogenetic analysis of α-1, 2-FTs was performed using MEGA7 [40] with 16 selected α-1, 2-FTs. A phylogenetic tree was constructed by the Maximum Likelihood method based on the JTT matrix model using two α-1, 3-FTs as a root. Positions containing alignment gaps and missing data were partially deleted. Statistical confidence of the inferred phylogenetic relationships was assessed by performing 1000 bootstrap replicates.

### Fed-batch fermentation

To produce 2′-FL by fed-batch fermentation, strain FL19 was first cultured in 5 mL SC medium containing glucose 2%, adenine 0.06 g/L and G418 250 µg/mL for 12 h. Then the culture broth was sub-cultured into a 100 mL shake flask containing 20 mL YP medium with 3% glucose and 0.12 g/L adenine for 24 h. Then 40 mL broth were inoculated into a 5 L fermenter (BioCore QF-5T, bolv, Shanghai) containing 2 L YP medium or the optimized Verduyn medium with glucose 4% (w/v), adenine (0.6 g/L). Sucrose 50% (w/v) and lactose 20% (w/v) were fed by a feeding pump to initial 2′-FL biosynthesis at 16 h after inoculum in YP medium. For the Verduyn medium, sucrose was fed 24 h after inoculum and lactose were fed at 36 h to initial 2′-FL biosynthesis. During the fermentation, temperature was kept at 30 °C and pH was controlled at 5.5 by feeding 3 M NaOH. The dissolved oxygen was kept constantly at 20%.

### Western blot analysis

Five milliliters of FL03 cells expressing His-tagged FutC or FutBc cultured on either glucose or galactose were harvested and washed twice with breaking buffer (50 mM sodium phosphate, pH 7.4, 1 mM phenylmethylsulfonyl fluoride, 1 mM EDTA, 5% glycerol) before being suspended with 1.5 ml breaking buffer. An equal volume of glass beads (0.5 mm, BioSpec) was added and the cells were disrupted in a homogenizer (Precellys 24, Bertin). The cellular extract was then centrifuged (10,000×*g*) for 10 min and the supernatant was used for western blot analysis. Western blot was performed as described [41] using an anti-His antibody (Sigma). The intensity of the blotted band was scanned with TOUCH IMAGER™ (E-BLOT, Shanghai, China) and quantified by calculating the Gray value.

## Supplementary Information

**Additional file 1: Table S1.** Primers used in this study. **Table S2.** Amino acid sequence identities (%) between α-1, 2-FTs tested in the present study and selected characterized α-1, 2-FTs in literatures. **Figure S1.** Lactose importing analysis by strain FL01 and FL02. **Figure S2.** Phylogenetic analysis and alignment of α-1, 2-FTs of different organisms including α-1, 2-FTs tested in the present study and selected α-1, 2-FTs reported in the literatures. **Figure S3.** Western blot assay of His-tagged FutBc and FutC expressed in yeast cells. **Figure S4.** 2′-FL production by FL06 with different initial lactose concentration. **Figure S5.** Cell wall stability test of strains producing 2′-FL. **Figure S6.** Effect of overexpression Pmi40 and sPos5 on 2′-FL production.

## Data Availability

The datasets used and/or analyzed during the current study are available from the corresponding author on reasonable request.

## Abbreviations

2′-FL: 2′‑fucosyllactose
FutBc: α-1, 2-fucosyltransferase from *Bacillus cereus*
HMOs: Human milk oligosaccharides
GDP-l-fucose: Guanosine 5′‑diphosphate‑L‑fucose
α-1, 2-FT: α-1,2-fucosyltransferase
GRAS: Generally recognized as safe
FutAs: α-1, 2-fucosyltransferase from *Acetobacter* sp.
FutBe: α-1, 2-fucosyltransferase from *B. eggerthii*
FutBu: α-1, 2-fucosyltransferase from *B. unifomis*
FutNc: α-1, 2-fucosyltransferase from *N. californiae*
GT11: Glycotransferase family 11
LC-MS: Liquid chromatography/mass spectrometry
NMR: Nuclear magnetic resonance
SDS: Sodium dodecyl sulfate
NADPH: Nicotinamide adenine dinucleotide phosphate
LB medium: Luria-Bertani medium
YPD medium: Yeast extract peptone dextrose medium
SC medium: Synthetic complete medium
TLC: Thin-layer chromatography
OD: Optical density
RI: Refractive index

## References

[1] Akira K (2010). Structures and application of oligosaccharides in human milk. P Jpn Acad B-Phys.

[2] Bode L (2012). Human milk oligosaccharides: every baby needs a sugar mama. Glycobiology.

[3] Koliwer-Brandl H, Siegert N, Umnus K, Kelm A, Tolkach A, Kulozik U, Kuballa J, Cartellieri S, Kelm S (2011). Lectin inhibition assays for the analysis of bioactive milk sialoglycoconjugates. Int Dairy J.

[4] Ruiz-Palacios GM, Luz Elena C, Pilar R, Bibiana CM, Newburg DS (2003). *Campylobacter jejuni* binds intestinal H(O) antigen (Fuc α 1, 2Gal β 1, 4GlcNAc), and fucosyloligosaccharides of human milk inhibit its binding and infection. J Biol Chem.

[5] Bing W, Bing Y, Muhsin K, Honghua H, Yun S, Paul MG, Peter P, Suzanne H, Jennie BM (2007). Dietary sialic acid supplementation improves learning and memory in piglets. Am J Clin Nutr..

[6] Phipps KR, Baldwin N, Lynch B, Flaxmer J, Soltesova A, Gilby B, Miks MH, Rohrig CH (2018). Safety evaluation of a mixture of the human-identical milk oligosaccharides 2'-fucosyllactose and difucosyllactose. Food Chem Toxicol.

[7] Agoston K, Hederos M, Bajza I, Dekany G (2019). Kilogram scale chemical synthesis of 2'-fucosyllactose. Carbohydr Res.

[8] Albermann C, Piepersberg W, Wehmeier UF (2001). Synthesis of the milk oligosaccharide 2'-fucosyllactose using recombinant bacterial enzymes. Carbohydr Res.

[9] Petschacher B, Nidetzky B (2016). Biotechnological production of fucosylated human milk oligosaccharides: Prokaryotic fucosyltransferases and their use in biocatalytic cascades or whole cell conversion systems. J Biotechnol.

[10] Lee W, Han N, Park Y, Seo J (2009). Modulation of guanosine 5'-diphosphate-D-mannose metabolism in recombinant *Escherichia coli* for production of guanosine 5'-diphosphate-l-fucose. Bioresour Technol.

[11] Coyne MJ, Barbara R, Lee MM, Comstock LE (2005). Human symbionts use a host-like pathway for surface fucosylation. Science.

[12] Baumgärtner F, Seitz L, Sprenger GA, Albermann C (2013). Construction of *Escherichia coli* strains with chromosomally integrated expression cassettes for the synthesis of 2'-fucosyllactose. Microb Cell Fact.

[13] Jung SM, Chin YW, Lee YG, Seo JH (2019). Enhanced production of 2'-fucosyllactose from fucose by elimination of rhamnose isomerase and arabinose isomerase in engineered emopenEscherichia coliemclose. Biotechnol Bioeng..

[14] Chin Y-W, Seo N, Kim JH, Seo JH (2016). Metabolic engineering of *Escherichia coli* to produce 2'-fucosyllactose via *salvage* pathway of guanosine 5'-diphosphate (GDP)-l-fucose. Biotechnol Bioeng.

[15] Nakayama K, Maeda Y, Jigami Y (2003). Interaction of GDP-4-keto-6-deoxymannose-3,5-epimerase-4-reductase with GDP-mannose-4,6-dehydratase stabilizes the enzyme activity for formation of GDP-fucose from GDP-mannose. Glycobiology.

[16] Huang D, Yang K, Liu J, Xu Y, Wang Y, Wang R, Liu B, Feng L (2017). Metabolic engineering of *Escherichia coli* for the production of 2'-fucosyllactose and 3'-fucosyllactose through modular pathway enhancement. Metab eng.

[17] Chin Y-W, Kim J-Y, Kim J-H, Jung S-M, Seo J-H (2017). Improved production of 2'-fucosyllactose in engineered *Escherichia coli* by expressing putative α-1, 2-fucosyltransferase, WcfB from *Bacteroides fragilis*. J Biotechnol.

[18] Deng J, Gu L, Chen T, Huang H, Yin X, Lv X, Liu Y, Li N, Liu Z, Li J (2019). Engineering the substrate transport and cofactor regeneration systems for enhancing 2'-fucosyllactose synthesis in *Bacillus subtilis*. ACS Synth Biol.

[19] Liu J, Kwak S, Pathanibul P, Lee J, Yu S, Yun EJ, Lim H, Kim KH, Jin Y-S (2018). Biosynthesis of a functional human milk oligosaccharide, 2'-Fucosyllactose, and l-fucose, using engineered emopenSaccharomyces cerevisiaeemclose. ACS Synth Biol.

[20] Hollands K, Baron CM, Gibson KJ, Kelly KJ, Krasley EA, Laffend LA, Lauchli RM, Maggio-Hall LA, Nelson MJ, Prasad JC (2019). Engineering two species of yeast as cell factories for 2'-fucosyllactose. Metab eng.

[21] Yu S, Liu J-J, Yun EJ, Kwak S, Kim KH, Jin Y-S (2018). Production of a human milk oligosaccharide 2'-fucosyllactose by metabolically engineered *Saccharomyces cerevisiae*. Microb Cell Fact.

[22] Orlean P (2012). Architecture and biosynthesis of the *Saccharomyces cerevisiae* cell wall. Genetics.

[23] Lee JW, Kwak S, Liu J-J, Yu S, Yun EJ, Kim DH, Liu C, Kim KH, Jin Y-S (2020). Enhanced 2'-Fucosyllactose production by engineered *Saccharomyces cerevisiae* using xylose as a co-substrate. Metab eng.

[24] Engels L, Elling L (2014). WbgL: a novel bacterial α1,2-fucosyltransferase for the synthesis of 2'-fucosyllactose. Glycobiology.

[25] Ban L, Pettit N, Li L, Stuparu AD, Cai L, Chen W, Guan W, Han W, Wang PG, Mrksich M (2012). Discovery of glycosyltransferases using carbohydrate arrays and mass spectrometry. Nat Chem Biol.

[26] Wang G, Boulton PG, Chan NWC, Palcic MM, Taylor DE (1999). Novel *Helicobacter pylori* α1,2-fucosyltransferase, a key enzyme in the synthesis of Lewis antigens. Microbiology.

[27] Shao J, Li M, Jia Q, Lu Y, Wang PG (2003). Sequence of *Escherichia coli* O128 antigen biosynthesis cluster and functional identification of an α-1,2-fucosyltransferase. FEBS Lett.

[28] Seydametova E, Yu J, Shin J, Park Y, Kim C, Kim H, Yu SH, Park Y, Kweon D (2019). Search for bacterial α1,2-fucosyltransferases for whole-cell biosynthesis of 2'-fucosyllactose in recombinant *Escherichia coli*. Microbiol Res.

[29] Urashima T, Hiramatsu Y, Murata S, Nakamura T, Messer M (1997). Identification of 2'-fucosyllactose in milk of the crabeater seal (*lobodon carcinophagus*). Comp Biochem Phys B.

[30] Pilauri V, Bewley M, Diep C, Hopper J (2005). Gal80 Dimerization and the Yeast GAL Gene Switch. Genetics.

[31] Lv X, Xie W, Lu W, Guo F, Gu J, Yu H, Ye L (2014). Enhanced isoprene biosynthesis in *Saccharomyces cerevisiae* by engineering of the native acetyl-CoA and mevalonic acid pathways with a push-pull-restrain strategy. J Biotechnol.

[32] Mattila P, Räbinä J, Hortling S, Helin J, Renkonen R (2000). Functional expression of *Escherichia coli* enzymes synthesizing GDP-l-fucose from inherent GDP-D-mannose in *Saccharomyces cerevisiae*. Glycobiology.

[33] Lee W, Shin S, Kim M, Han NS, Seo J (2012). Modulation of guanosine nucleotides biosynthetic pathways enhanced GDP-l-fucose production in recombinant *Escherichia coli*. Appl Microbiol Biotechnol.

[34] Lee WH, Kim MD, Jin Y, Seo J (2013). Engineering of NADPH regenerators in *Escherichia coli* for enhanced biotransformation. Appl Microbiol Biotechnol.

[35] Sauer U, Canonaco F, Heri S, Perrenoud A, Fischer E (2004). The soluble and membrane-bound transhydrogenases UdhA and PntAB have divergent functions in NADPH metabolism of *Escherichia coli*. J Biol Chem.

[36] Wang B, Wang P, Zheng E, Chen X, Zhao H, Song P, Su R, Li X, Zhu G (2011). Biochemical properties and physiological roles of NADP-dependent malic enzyme in *Escherichia coli*. J Microbiol.

[37] van Hoek P, de Hulster E, van Dijken JP, Pronk JT (2000). Fermentative capacity in high-cell-density fed-batch cultures of baker's yeast. Biotechnol Bioeng.

[38] Xia Y, Li K, Li J, Wang T, Gu L, Xun L (2018). T5 exonuclease-dependent assembly offers a low-cost method for efficient cloning and site-directed mutagenesis. Nucleic Acids Res.

[39] Gietz RD, Schiestl RH (2007). High-efficiency yeast transformation using the LiAc/SS carrier DNA/PEG method. Nat Protoc.

[40] Kumar S, Stecher G, Tamura K (2016). MEGA7: molecular evolutionary genetics analysis version 7.0 for bigger datasets. Mol Biol Evol.

[41] Blancher C, Jones A. SDS-PAGE and western blotting techniques. In: Brooks SA, Schumacher U, editors. Metastasis research protocols: volume I: analysis of cells and tissues*.* Totowa: Humana Press; 2001, pp 145–62. 10.1385/1-59259-136-1:145 21340897

