# Supplementary material for: Improved production of 2′-fucosyllactose in engineered Saccharomyces cerevisiae expressing a putative α-1, 2-fucosyltransferase from Bacillus cereus
Source: Microb Cell Fact. 2021 Aug 23;20:165. doi: 10.1186/s12934-021-01657-5 (PMC8381501; doi:10.1186/s12934-021-01657-5)
Supplement: Supplementary file 1 — Additional file 1: Table S1. Primers used in this study. Table S2. Amino acid sequence identities (%) between α-1, 2-FTs tested in the present study and selected characterized α-1, 2-FTs in literatures. Figure S1. Lactose importing analysis by strain FL01 and FL02. Figure S2. Phylogenetic analysis and alignment of α-1, 2-FTs of different organisms including α-1, 2-FTs tested in the present study and selected α-1, 2-FTs reported in the literatures. Figure S3. Western blot assay of His-tagged FutBc and FutC expressed in yeast cells. Figure S4. 2′-FL production by FL06 with different initial lactose concentration. Figure S5. Cell wall stability test of strains producing 2′-FL. Figure S6. Effect of overexpression Pmi40 and sPos5 on 2′-FL production. [file 12934_2021_1657_MOESM1_ESM.docx]

**Supplementary materials**

**Improved production of 2′-fucosyllactose in** **engineered *Saccharomyces cerevisiae* expressing a putative α-1, 2-fucosyltransferase from *Bacillus cereus***

**Mingyuan Xu, Xiangfeng Meng**^*^**, Weixin Zhang, Yu Shen, Weifeng Liu**^*^

State Key Laboratory of Microbial Technology, Microbial Technology Institute, Shandong University, No.72 Binhai Road, Qingdao 266237, P. R. China

^*^Correspondence should be addressed to X Meng and W Liu. E-mail: x.meng@sdu.edu.cn; weifliu@sdu.edu.cn; Phone: +86 0532 58632405; Fax: +86 0532 58631501

Table S1 Primers used in this study

| **Name** | **Sequence** | **Description** |
| --- | --- | --- |
| BamHI-GAL1-F | GCTCTAGAACTAGTGCGCGGATCCAGTACGGATTA GAAG | Assemble of *lac12* expression cassette in pRS304 with an inducible promoter P*_gal1_* and terminator T*_cyc1_* |
| GAL1-LAC12-R | GATCTGCCATGGGTTTTTTCTC CTT |  |
| gal1-LAC12-F | GAAAAAACCCATGGCAGATCA TTCG |  |
| LAC12-cyc1-R | AATTACATGATTAAACAGATTCTGCCTC |  |
| lac12-CYC1-F | ATCTGTTTAATCATGTAATTAGTTATGTCACG |  |
| CYC1-XhoI-R | GTACCGGGCCCCCCCCCGCTCGAGGCAAATTAAAG CCTT |  |
| XhoI-GAL1-F | GTACCGGGCCCCCCCCCGCTCGAGAGTACGGATTAGAAGCCGCC | Assemble of *lac4* expression cassette in pRS425 with an inducible promoter P*_gal1_* and terminator T*_cyc1_* |
| GAL1-LAC4-R | GGCAAGACATGGGTTTTTTCTCCTT |  |
| gal1-LAC4-F | GAAAAAACCCATGTCTTGCCTTATTCCTGAGAAT |  |
| LAC4-cyc1-R | AATTACATGATTATTCAAAAGCGAGATCAAACTCA |  |
| lac4-CYC1-F | AATAATCATGTAATTAGTTATGTCACGCTTAC |  |
| CYC1-SpeI-R | GCGGCCGCTCTAGAACTAGACTAGTGCAAATTAAAGCCTT CGAGC |  |
| SacI-ADH1-F | GGGCGAATTGGAGCTCGAGCTCAGCTGAATTGGAGCGACCTCAT | Assemble of *wcaG* and *gmd* gene with a bi-directional expression cassette amplified from pUMRI-A resulting a plasmid, in which *wcaG* and *gmd* were expressed under *P_gal1_* and P*_gal10_*, respectively. |
| ADH1-wcaG-R | GGGGTAAGCCAGAGGTTTGGTC |  |
| adh-WcaG-F | CTCTGGCTTACCCCCGAAAGCGGTCTT |  |
| WcaG-10-R | CGACGATAAGATGAGTAAACAACGAGTTTTTATTGCTG |  |
| wcag-10-F | ACTCATCTTATCGTCGTCATCCTTG |  |
| 10-gmd-R | CTTTTGACATGAGGTCTTCTTCGGA |  |
| 10-Gmd-F | ACCTCATGTCAAAAGTCGCTCTCATCAC |  |
| Gmd-cyc-R | CGGATTTATGACTCCAGCGCGATC |  |
| gmd-CYC1-F | GGAGTCATAAATCCGCTCTAACCGA |  |
| CYC1-KpnI-R | ACAAAAGCTGGGTACCGGGGTACCCTTCGAGCGTCCCAAAACCTT |  |
| XbaI-Gal1-F | CGCGGTGGCGGCCGCGCTCTAGAAGTACGGATTA GAAG | Assemble the expression cassette of FutC from *H. pylori* in pRS305 |
| Gal1-futC-R | TAAATGCCAT GGGTTTTTTC TCCTT |  |
| gal1-FutC-F | GAAAAAACCC ATGGCATTTAAAGTG |  |
| FutC-cyc1-R | AATTACATGACTAAGCATTATATTTCTG |  |
| futC-CYC1-F | ATATAATGCTTAGTCATGTAATTAGTTATGTCACG |  |
| HindIII-CYC1-R | GTCGACGGTATCGATCCCAAGCTTGCAAATTAAAGCCTT |  |
| Gal1 BC-R | TAATCTTCAT GGGTTTTTTCTCCTTG | Assemble the expression cassette of FutBc from *B. cereus* in pRS305 |
| Fut BC-F | GAAAAAACCCATGAAGATTATTCAGGTG |  |
| fut-BC-R | AATTACATGATCAATAAGTAATCCAATT |  |
| BC CYC1-F | TACTTATTGATCATGTAATTAGTTATGTCACG |  |
| Gal1 BU-R | CAATTTTCAT GGGTTTTTTCTCCTTG | Assemble the expression cassette of FutBu from *B. unifomis* in pRS305 |
| Fut BU-F | GAAAAAACCCATGAAAATTGTGCTGCCA |  |
| fut-BU-R | AATTACATGATCACATTCTAATCCATTC |  |
| BU CYC1-F | TAGAATGTGATCATGTAATTAGTTATGTCACG |  |
| Gal1 BE-R | TCAGTCTCAT GGGTTTTTTCTCCTTG | Assemble the expression cassette of FutBe from *B. eggerthii* in pRS305 |
| Fut BE-F | GAAAAAACCCATGAGACTGATTAAAATG |  |
| fut-BE-R | AATTACATGATCAATTAACTGGAACTTT |  |
| BE CYC1-F | AGTTAATTGATCATGTAATTAGTTATGTCACG |  |
| Gal1 NC-R | GAATTTTCATGGGTTTTTTCTCCTTG | Assemble the expression cassette of FutNc from *N. californiae* in pRS305 |
| Fut NC-F | GAAAAAACCCATGAAAATTCTGATTTTT |  |
| fut-NC-R | AATTACATGATCATGAAGAATTATTAGA |  |
| NC CYC1-F | TTCTTCATGATCATGTAATTAGTTATGTCACG |  |
| XbaI-ADH1-F | CACCGCGGTGGCGGCCGCTCTAGAAGCTGAATTGGAGCGACCTCAT | Assemble of *sec53* and *psa1* gene with a bi-directional expression cassette amplified from pUMRI-A, resulting a plasmid, in which *sec53* and *psa1* were expressed under *P_gal1_* and P*_gal10_*, respectively. |
| ADH1-sec-R | CAACTTATAGGCCAGAGGTTTGGTCAAGTCTC |  |
| SEC-F | AAACCTCTGGCCTATAAGTTGAATAGTTCAGTC |  |
| 10-SEC-R | CTAGTATCGATGAGTATCGCTGAATTCGCTTAC |  |
| 10-s-F | CAGCGATACTCATCGATACTAGTGCGGCCGCCC |  |
| 10-p-R | AAACCTTTCATGTCGACGCCCGGGCCCTATAG |  |
| 10-PSA2-F | GGCGTCGACATGAAAGGTTTAATTTTAGTCGG |  |
| PSA2-R | GTTAGAGCGGATTCACATAATAATAGCTTCC |  |
| CYC1-psa2-F | TTATGTGAATCCGCTCTAACCGAAAAGGAAGG |  |
| EcoRI-cyc1-R | CGATAAGCTTGATATCGAATTCCTTCGAGCGTCCCAAAACCTTC |  |
| BamHI-pmi40-F | AAGGAGAAAAAACCCCGGATCCATGTCCAACAAGCTGTTCAG | *pmi40* expression cassette construction in pUMRI-A |
| NheI-pmi40-R | GTTAGAGCGGATCTTAGCTAGCCTAATTTGGTTCCACAAAGG |  |
| EcoRI-sPos5-F | GAATTTTTGAAAATTCGAATTCATGAGTACGTTGGATTCACATTC | *spos5* expression cassette construction in pUMRI-A |
| SacI-sPos5-R | AGAATTGTTAATTAAGAGCTCTTAATCATTATCAGTCTGTCTCTTGGTC |  |
| Gal80-knockout-F | GTATACAATCTCGATAGTTGGTTTCCCGTTCTTTCCACTCCCGTCTAACTTCGTATAATGTATGC | Gal80 knockout cassette |
| Gal80-knockout-R | TTACCCACAATGGCATTATAATTTCGTAAATGATATACTTCCATGATAACTTCGTATAGCATAC |  |

Restriction sites are underlined.

Table S2 Amino acid sequence identities (%) between α-1, 2-FTs tested in the present study and selected characterized α-1, 2-FTs

| FutF | 16.81 |  |  |  |  |  |  |  |  |  |  |  |  |  |  |  |  |
| --- | --- | --- | --- | --- | --- | --- | --- | --- | --- | --- | --- | --- | --- | --- | --- | --- | --- |
| FutG | 18.07 | 46.35 |  |  |  |  |  |  |  |  |  |  |  |  |  |  |  |
| WcfW | 23.04 | 17.98 | 18.25 |  |  |  |  |  |  |  |  |  |  |  |  |  |  |
| FutNc | 17.60 | 19.92 | 19.38 | 26.14 |  |  |  |  |  |  |  |  |  |  |  |  |  |
| FutC | 19.75 | 17.83 | 20.87 | 25.10 | 37.31 |  |  |  |  |  |  |  |  |  |  |  |  |
| FutL | 21.40 | 20.65 | 20.08 | 26.69 | 39.31 | 73.43 |  |  |  |  |  |  |  |  |  |  |  |
| WcfB | 19.92 | 15.89 | 18.04 | 26.97 | 28.40 | 26.24 | 28.02 |  |  |  |  |  |  |  |  |  |  |
| WbiQ | 20.00 | 18.68 | 17.97 | 33.47 | 31.56 | 28.36 | 25.95 | 25.91 |  |  |  |  |  |  |  |  |  |
| WbwK | 16.53 | 17.56 | 16.92 | 31.30 | 31.18 | 29.70 | 27.69 | 24.36 | 49.66 |  |  |  |  |  |  |  |  |
| WbgL | 20.00 | 18.85 | 21.88 | 29.55 | 30.53 | 25.00 | 22.90 | 24.64 | 29.24 | 28.99 |  |  |  |  |  |  |  |
| WbsJ | 20.00 | 24.00 | 20.88 | 35.68 | 32.02 | 30.50 | 29.25 | 29.01 | 30.37 | 32.10 | 31.20 |  |  |  |  |  |  |
| FutAs | 23.55 | 18.99 | 17.05 | 31.05 | 31.29 | 33.21 | 33.20 | 30.51 | 28.00 | 28.99 | 29.64 | 34.72 |  |  |  |  |  |
| FutBc | 21.49 | 24.71 | 24.41 | 31.40 | 34.90 | 29.34 | 32.81 | 28.73 | 30.91 | 30.43 | 32.38 | 33.33 | 32.73 |  |  |  |  |
| FutTe | 24.38 | 23.26 | 20.62 | 29.96 | 29.96 | 31.06 | 33.33 | 31.79 | 28.42 | 31.29 | 29.93 | 30.51 | 32.86 | 36.36 |  |  |  |
| FutBu | 24.58 | 20.83 | 19.01 | 32.39 | 29.30 | 31.42 | 32.16 | 25.64 | 27.27 | 26.55 | 27.54 | 30.60 | 32.00 | 37.99 | 37.59 |  |  |
| FutBe | 25.42 | 22.40 | 21.86 | 29.11 | 31.23 | 30.86 | 30.00 | 27.51 | 28.79 | 29.66 | 29.35 | 34.77 | 31.23 | 38.10 | 38.41 | 38.97 |  |
| FutN | 22.76 | 22.83 | 23.02 | 28.69 | 30.42 | 29.46 | 30.95 | 31.23 | 29.17 | 28.90 | 29.35 | 35.94 | 30.47 | 38.46 | 36.59 | 39.34 | 75.80 |
|  | FucT2 | FutF | FutG | WcfW | FutNc | FutC | FutL | WcfB | WbiQ | WbwK | WbgL | WbsJ | FutAs | FutBc | FutTe | FutBu | FUtBe |


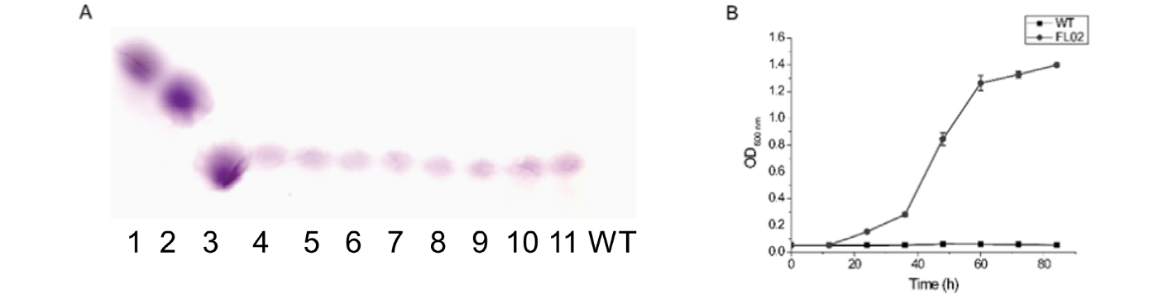


Figure S1. Lactose importing analysis of strain FL01 and FL02. (A) TLC analysis of the intracellular accumulation of lactose in FL01. Lane 1: glucose; Lane 2: galactose; Lane 3: lactose; Lane 4-11: intracellular lactose of different transformants of FL01. WT: *S. cerevisiae* W303-1a (WT) control. (B) Growth curve of FL02 and *S. cerevisiae* W303-1a (WT) in YP medium with lactose as the sole carbon source.


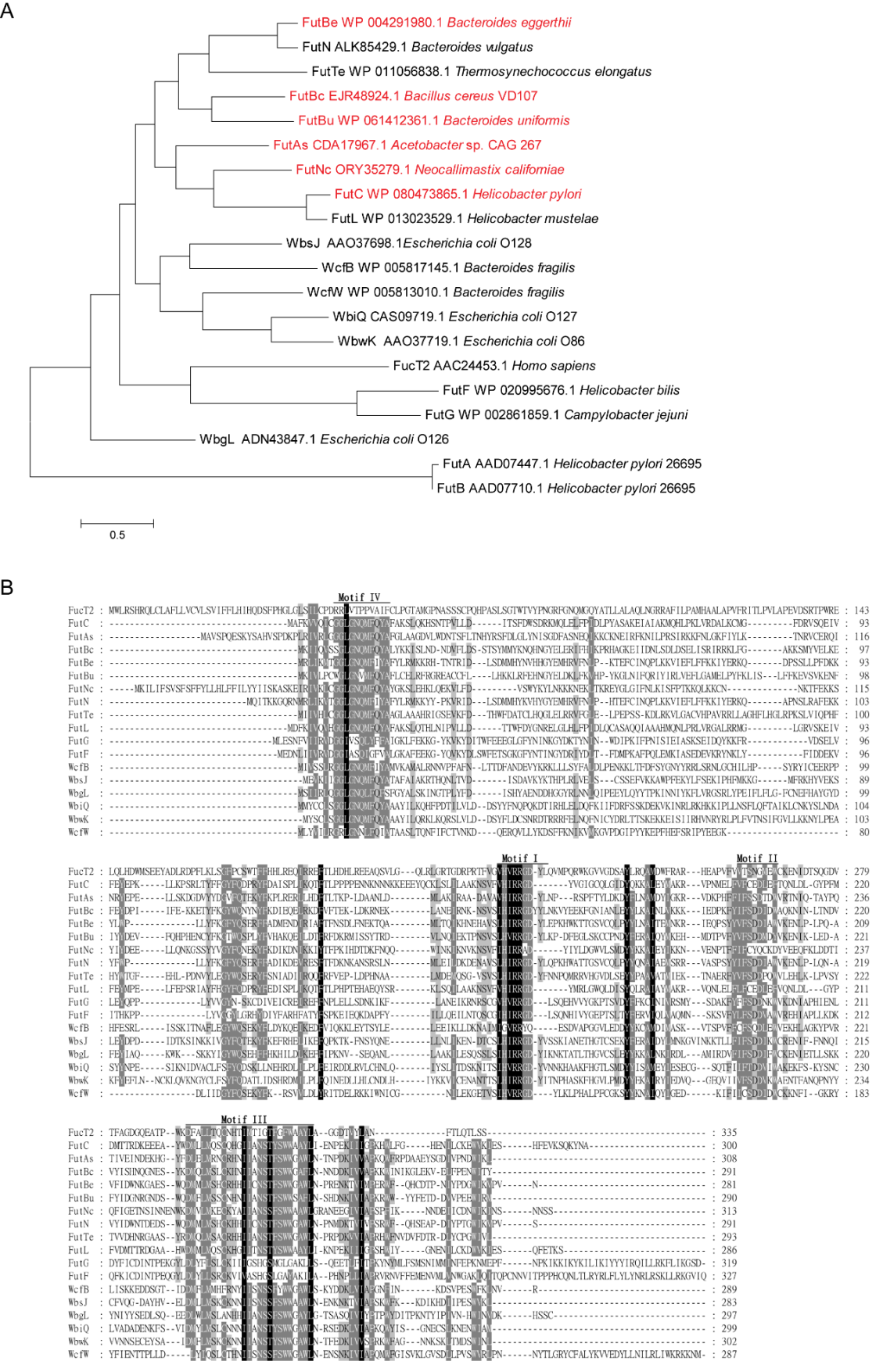


Figure S2. Phylogenetic analysis (A) and alignment (B) of α-1, 2-FTs from different organisms including α-1, 2-FTs tested in the present study (in red) and selected α-1, 2-FTs reported in the literatures. α-1, 3-FTs FutA and FutB of *H. pylori* 26695 are used as roots for the phylogenetic analysis. The alignments were performed with Clustal W.


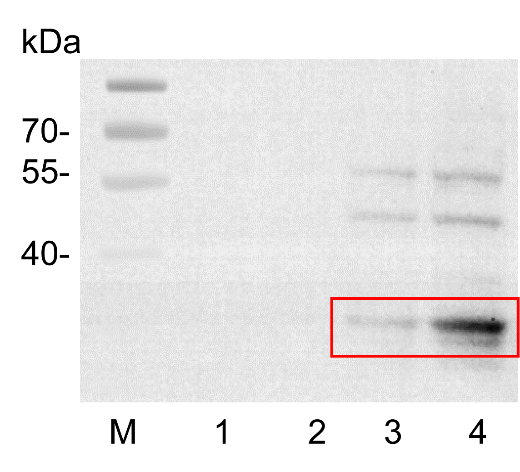


Figure S3. Western blot analysis of His-tagged FutBc and FutC in yeast cells. Band in red box showed the respective western blot signal of FutC and FutBC. M, marker; 1, FutC cultured in YP with glucose (2% w/v); 2, FutBc cultured in YP with glucose (2% w/v); 3, FutC cultured in YP with glucose (2% w/v) and galactose (2% w/v); 4, FutBc cultured in YP with glucose (2% w/v) and galactose (2% w/v); The total protein was extracted from 5 mL of yeast cells culture and homogenized in breaking buffer (50 mM sodium phosphate, pH 7.4, 1 mM phenylmethylsulfonyl fluoride, 1 mM EDTA, 5% glycerol).


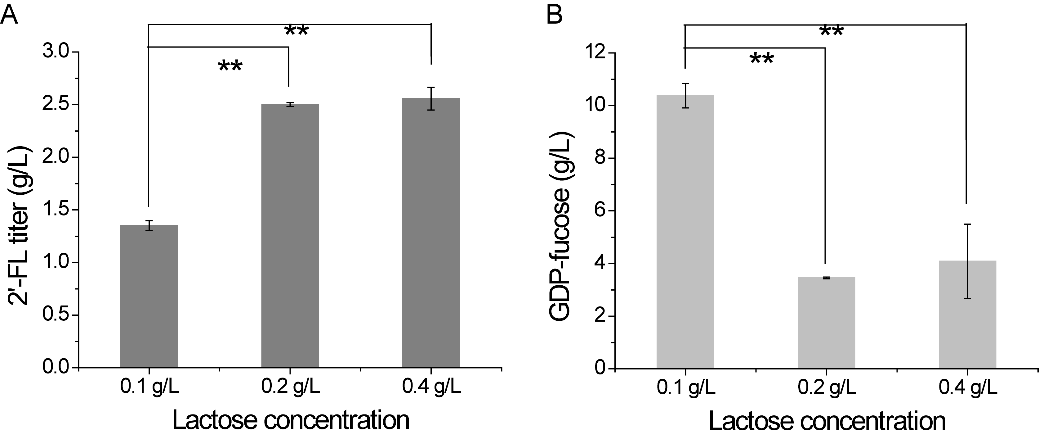


Figure S4. 2’-FL production by FL06 under different initial lactose concentrations. The strain was cultured in YP medium with 0.1%, 0.2% or 0.4% (w/v) lactose, respectively. (A) 2’-FL production under different lactose concentrations. (B) GDP-L-fucose concentration under different lactose concentrations (t test, * *p* < 0.05, ** *p* < 0.01).


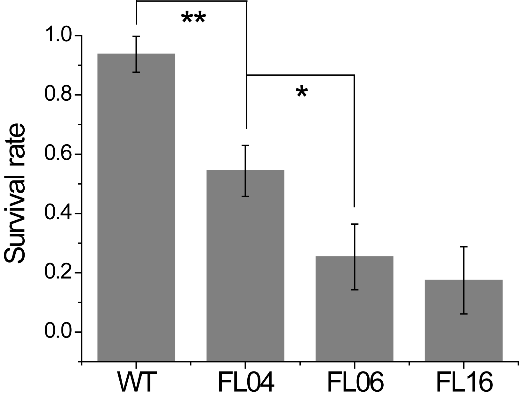


Figure S5. Cell wall stability test for strains producing 2’-FL. Cells from culture (1 mL) at 72 h were collected, washed and resuspended in 10 mM HEPES buffer (pH 7.5) and 10 mM HEPES buffer (pH 7.5) with 0.1% SDS, respectively. After incubating at room temperature for 20 min, the cells were diluted (10^6^-fold) and then spread on SC plate. After cultured at 30^o^C for 48 h, Survival rate was calculated by dividing the number of colonies with SDS treatment by that of without SDS treatment. Significant differences were observed for the cell wall stability between the wild type strain and F04 as well as between F04 and F06 (t test, * *p* < 0.05, ** *p* < 0.01).


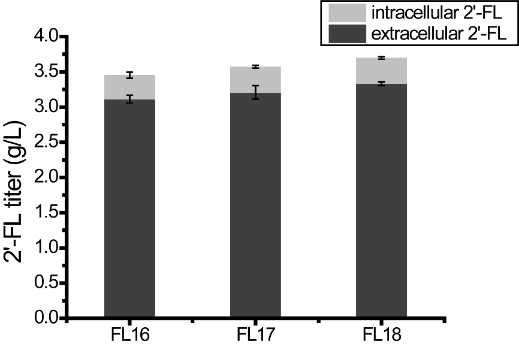


Figure S6. Effects of overexpression Pmi40 (FL17) and sPos5 (FL18) on 2’-FL production. FL17 is constructed by additional overexpression of Pmi40 in FL16. FL18 is constructed by additional overexpression of sPos5 in FL17.
